# Supplementary material for: Regression-based modeling of pairwise genomic linkage data identifies risk factors for healthcare-associated pathogen transmission: application to carbapenem-resistant Klebsiella pneumoniae transmission in a long-term care facility
Source: Microbiol Spectr. 2026 Jan 12;14(2):e02452-25. doi: 10.1128/spectrum.02452-25 (PMC12889109; doi:10.1128/spectrum.02452-25)
Supplement: Supplemental tables and figure — Tables S1 to S4 and Figure S1. [file spectrum.02452-25-s0001.pdf]

Supplemental Table S1. Results of pairwise regression models with shared floor as a single covariate (n = 174 patients, 3500 pairs). Model 1 is a log-linear model of pairwise SNV distance as a function of individual and pairwise exposure risks. Coefficients are exponentiated and can be interpreted analogously to rate ratios, with values < 1 indicating smaller distances and > 1 indicating greater distances. Models 2 & 3 are logistic regression models characterizing changes in the odds that a given infectious case is the most closely related to the recipient (Model 2) or that the infectious case and exposed individual are in the same genomic cluster (Model 3). All results are adjusted for all covariates in the model, and a random effect for potential donor is included in the models.

	Model 1. SNV Distance Model	Model 2. Closest Donor Model	Model 3. Same Cluster Model
Intercept	**49.66 (44.34, 55.61)**	**0.02 (0.01, 0.04)**	**0.01 (0.00, 0.03)**
Shared Floor[1]	**0.92 (0.88, 0.97)**	**2.10 (1.41, 3.14)**	**2.72 (1.82, 4.06)**
Shared Room[1]	**0.74 (0.59, 0.92)**	2.91 (0.93, 9.11)	**3.99 (1.18, 13.45)**
Culture Date Difference (per 30 days)	1.02 (1.00, 1.04)	**0.85 (0.75, 0.96)**	**0.68 (0.58, 0.79)**
Carbapenem antibiotic exposure of donor[1]	1.04 (0.97, 1.10)	1.05 (0.65, 1.70)	0.78 (0.46, 1.32)
Non-Carbapenem antibiotic exposure of donor[1]	1.03 (0.96, 1.11)	0.69 (0.39, 1.23)	0.89 (0.48, 1.63)
Time period (quarter 2 v quarter 1)[2]	0.95 (0.87, 1.04)	1.38 (0.67, 2.81)	1.80 (0.80, 4.04)
Time period (quarter 3 v quarter 1)[2]	0.98 (0.88, 1.10)	1.08 (0.50, 2.34)	2.02 (0.86, 4.75)
Time period (quarter 4 v quarter1)[2]	**0.87 (0.76, 0.99)**	1.52 (0.68, 3.37)	**4.42 (1.84, 10.61)**

Estimates are exponentiated and 95% confidence intervals are in parentheses. Bolded values have confidence intervals that do not contain 1.

[1]During pairwise exposure period

[2]When recipient first tested positive

SNV = single nucleotide variant.

Supplemental Table S2. Drivers of variation in pairwise genomic relatedness as a function of individual and pair-level risk factors including pairs that did not overlap in the facility (n = 174 patients, 10,931 pairs). Model 1 is a log-linear model of pairwise SNV distance as a function of individual and pairwise exposure risks. Coefficients are exponentiated and can be interpreted analogously to rate ratios, with values < 1 indicating smaller distances and > 1 indicating greater distances. Models 2 & 3 are logistic regression models characterizing changes in the odds that a given infectious case is the most closely related to the recipient (Model 2) or that the infectious case and exposed individual are in the same genomic cluster (Model 3). All results are adjusted for all covariates in the model, and a random effect for potential donor is included in the models.

	Model 1. SNV Distance Model	Model 2. Closest Donor Model	Model 3. Same Cluster Model
Intercept	**52.27 (45.72, 59.76)**	**0.01 (0.00, 0.02)**	**0.00 (0.00, 0.01)**
Shared Floor A[1]	**0.81 (0.74, 0.90)**	**4.53 (1.58, 13.03)**	**3.36 (1.23, 9.20)**
Shared Floor B[1]	0.99 (0.95, 1.03)	1.79 (0.99, 3.25)	1.41 (0.83, 2.38)
Shared Floor C[1]	1.05 (0.97, 1.12)	1.58 (0.62, 4.04)	1.46 (0.59, 3.59)
Shared Floor D[1] (Includes High Acuity Unit)	**0.59 (0.55, 0.64)**	**9.76 (5.09, 18.74)**	**9.27 (5.54, 15.52)**
Shared Floor E[1]	**1.31 (1.11, 1.54)**	2.27 (0.25, 20.32)	0.46 (0.04, 5.00)
Shared room[1]	**0.72 (0.60, 0.86)**	**4.57 (1.18, 17.63)**	3.86 (0.91, 16.30)
Culture date difference (30 days)	**1.01 (1.00, 1.02)**	**0.79 (0.72, 0.87)**	**0.65 (0.59, 0.72)**
Carbapenem antibiotic exposure of donor[2]	0.96 (0.90, 1.02)	1.30 (0.72, 2.34)	1.59 (0.89, 2.85)
Non-Carbapenem antibiotic exposure of donor[2]	1.00 (0.88, 1.13)	0.84 (0.29, 2.43)	1.93 (0.60, 6.16)
Time period (quarter 2 v quarter 1)[3]	0.95 (0.90, 1.00)	1.30 (0.65, 2.62)	3.20 (1.58, 6.46)
Time period (quarter 3 v quarter 1)[3]	1.03 (0.96, 1.10)	1.06 (0.49, 2.28)	3.71 (1.69, 8.15)
Time period (quarter 4 v quarter1)[3]	0.92 (0.84, 1.00)	1.61 (0.70, 3.71)	**6.22 (2.62, 14.75)**

Estimates are exponentiated and 95% confidence intervals are in parentheses. Bolded values have confidence intervals that do not contain 1.

[1]During pairwise exposure period

[2]During period between when the donor last tested negative and the recipient tested positive

[3]When recipient first tested positive

SNV = single nucleotide variant.

Supplemental Table 3. Drivers of variation in pairwise genomic relatedness as a function of individual and pair-level risk factors including pairs that did not overlap in the facility and successive room sharing variables (n = 174 patients, 10,931 pairs). Model 1 is a log-linear model of pairwise SNV distance as a function of individual and pairwise exposure risks. Coefficients are exponentiated and can be interpreted analogously to rate ratios, with values < 1 indicating smaller distances and > 1 indicating greater distances. Models 2 & 3 are logistic regression models characterizing changes in the odds that a given infectious case is the most closely related to the recipient (Model 2) or that the infectious case and exposed individual are in the same genomic cluster (Model 3). All results are adjusted for all covariates in the model, and a random effect for potential donor is included in the models.

	Model 1. SNV Distance Model	Model 2. Closest Donor Model	Model 3. Same Cluster Model
Intercept	**52.22 (45.67, 59.71)**	**0.01 (0.00, 0.02)**	**0.00 (0.00, 0.01)**
Shared Floor A[1]	**0.81 (0.73, 0.89)**	**5.11 (1.76, 14.83)**	**3.19 (1.15, 8.83)**
Shared Floor B[1]	0.98 (0.94, 1.02)	**1.86 (1.02, 3.38)**	1.40 (0.82, 2.37)
Shared Floor C[1]	1.04 (0.97, 1.12)	1.62 (0.63, 4.17)	1.46 (0.59, 3.60)
Shared Floor D[1] (Includes High Acuity Unit)	**0.59 (0.55, 0.64)**	**9.94 (5.16, 19.15)**	**9.21 (5.50, 15.43)**
Shared Floor E[1]	**1.31 (1.11, 1.54)**	2.27 (0.25, 20.37)	0.45 (0.04, 4.89)
Shared room			
During pairwise exposure period	**0.72 (0.60, 0.86)**	**4.47 (1.15, 17.29)**	4.00 (0.94, 16.94)
1-7 days before pairwise exposure period	1.11 (0.96, 1.28)	0.93 (0.11, 7.63)	1.08 (0.14, 8.62)
7-30 days before pairwise exposure period	1.06 (0.96, 1.16)	0.48 (0.10, 2.25)	0.86 (0.24, 3.05)
31-90 days before pairwise exposure period	1.01 (0.94, 1.09)	0.63 (0.17, 2.34)	1.75 (0.74, 4.10)
91 days-1 year before pairwise exposure period	0.99 (0.94, 1.04)	1.33 (0.51, 3.47)	1.82 (0.79, 4.20)
Culture date difference (30 days)	**1.01 (1.00, 1.02)**	**0.79 (0.71, 0.87)**	**0.65 (0.58, 0.72)**
Carbapenem antibiotic exposure of donor[2]	0.96 (0.90, 1.02)	1.31 (0.73, 2.37)	1.58 (0.88, 2.83)
Non-Carbapenem antibiotic exposure of donor[2]	1.00 (0.88, 1.13)	0.84 (0.29, 2.45)	1.94 (0.61, 6.19)

Time period (quarter 2 v quarter 1)[3]	0.95 (0.90, 1.00)	1.32 (0.65, 2.67)	**3.08 (1.52, 6.22)**
Time period (quarter 3 v quarter 1)[3]	1.03 (0.96, 1.10)	1.06 (0.49, 2.29)	**3.61 (1.64, 7.92)**
Time period (quarter 4 v quarter1)[3]	0.92 (0.84, 1.00)	1.58 (0.68, 3.67)	**5.98 (2.52, 14.19)**

Estimates are exponentiated and 95% confidence intervals are in parentheses. Bolded values have confidence intervals that do not contain 1.

[1]During pairwise exposure period

[2]During period between when the donor last tested negative and the recipient tested positive

[3]When recipient first tested positive

SNV = single nucleotide variant.

Supplemental Table S4. Results of pairwise regression models excluding individuals who did not have a positive colonization isolate on admission or a CRKP isolate detected via clinical testing during the study period (n = 121 patients, 1354 pairs). Model 1 is a log-linear model of pairwise SNV distance as a function of individual and pairwise exposure risks. Coefficients are exponentiated and can be interpreted analogously to rate ratios, with values < 1 indicating smaller distances and > 1 indicating greater distances. Models 2 & 3 are logistic regression models characterizing changes in the odds that a given infectious case is the most closely related to the recipient (Model 2) or that the infectious case and exposed individual are in the same genomic cluster (Model 3). All results are adjusted for all covariates in the model, and a random effect for potential donor is included in the models.

	Model 1. SNV Distance Model	Model 2. Closest Donor Model	Model 3. Same Cluster Model
Intercept	**51.00 (43.35, 60.01)**	**0.03 (0.01, 0.10)**	**0.01 (0.00, 0.04)**
Shared Floor[1]	0.97 (0.91, 1.04)	1.57 (0.85, 2.89)	1.29 (0.63, 2.61)
Shared Room[1]	0.90 (0.67, 1.22)	2.07 (0.34, 12.61)	2.40 (0.21, 27.72)
Culture Date Difference (per 30 days)	**1.03 (1.01, 1.05)**	**0.84 (0.71, 0.99)**	**0.74 (0.60, 0.91)**
Carbapenem antibiotic exposure of donor[1]	0.97 (0.89, 1.07)	1.70 (0.84, 3.43)	1.52 (0.64, 3.65)
Non-Carbapenem antibiotic exposure of donor[1]	1.01 (0.89, 1.15)	0.41 (0.17, 1.02)	0.84 (0.27, 2.60)
Time period (quarter 2 v quarter 1)[2]	1.06 (0.94, 1.20)	1.07 (0.36, 3.18)	1.20 (0.25, 5.66)
Time period (quarter 3 v quarter 1)[2]	0.93 (0.80, 1.09)	1.21 (0.40, 3.66)	2.61 (0.58, 11.77)
Time period (quarter 4 v quarter1)[2]	**0.82 (0.69, 0.98)**	2.16 (0.74, 6.29)	**7.83 (1.78, 34.39)**

Estimates are exponentiated and 95% confidence intervals are in parentheses. Bolded values have confidence intervals that do not contain 1.

[1]During pairwise exposure period

[2]When recipient first tested positive

SNV = single nucleotide variant.

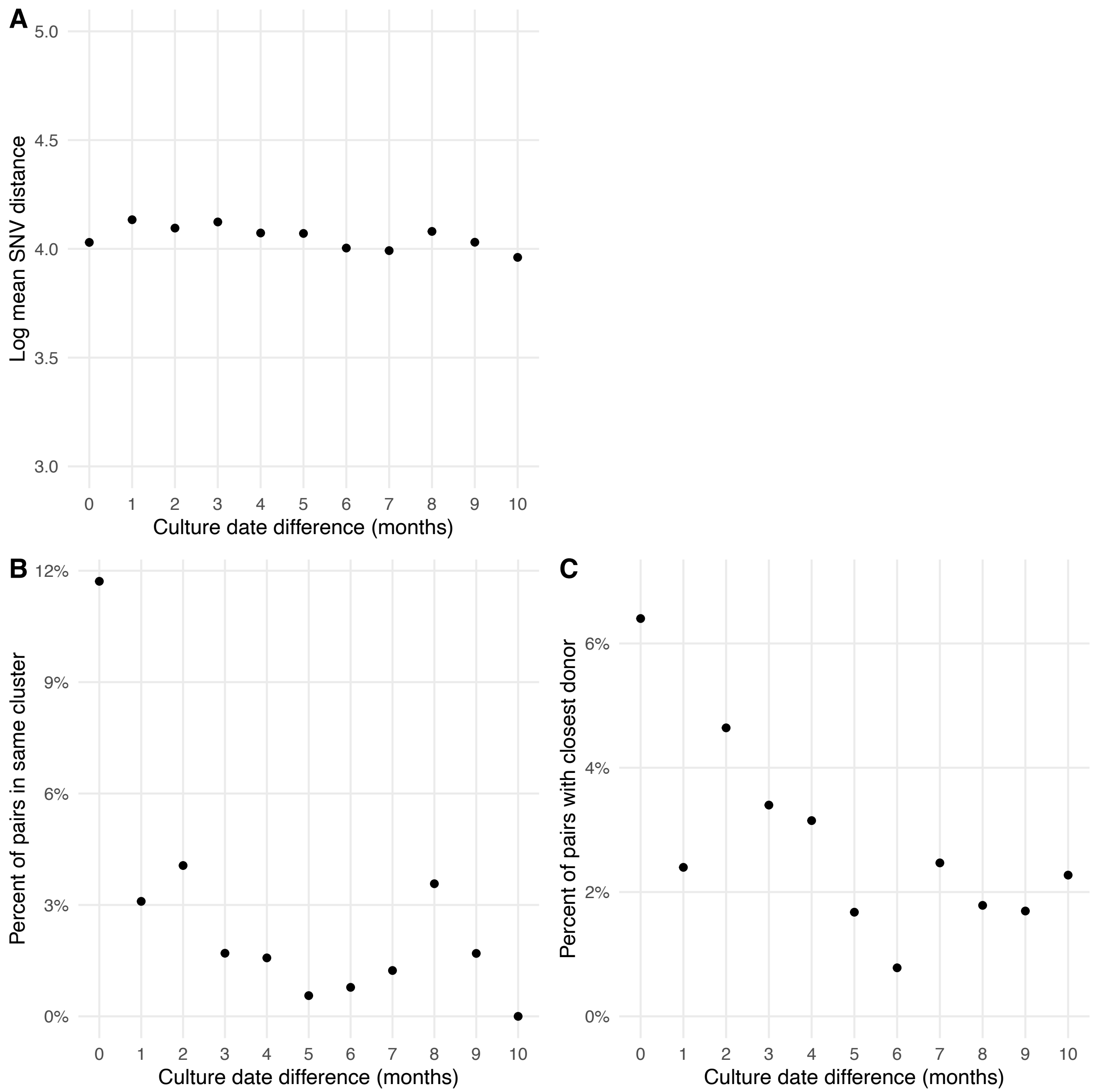

Supplemental Figure S1. Relationship between pairwise culture date difference and model outcomes. Culture date difference appears to **(A)** not be associated with log mean single nucleotide variant (SNV) distance, and to have a log linear negative relationship with **(B)** pairs in the same cluster and **(C)** pairs that include the recipient's closest donor. This satisfies the assumptions of log-linear relationships between the linear predictor and outcome in our pairwise regression models. Due to low sample size, culture date differences of ten or more months are categorized as ten months (maximum culture date difference = 12 months).
